# Contextual Positive Psychology: Policy Recommendations for Implementing Positive Psychology into Schools

**DOI:** 10.3389/fpsyg.2016.01561

**Published:** 2016-10-10

**Authors:** Joseph Ciarrochi, Paul W. B. Atkins, Louise L. Hayes, Baljinder K. Sahdra, Philip Parker

**Affiliations:** ^1^Institute for Positive Psychology and Education, Australian Catholic University, SyndeyNSW, Australia; ^2^Orygen, The National Centre of Excellence in Youth Mental Health, MelbourneVIC, Australia; ^3^Centre for Youth Mental Health, The University of Melbourne, MelbourneVIC, Australia

**Keywords:** psychological flexibility, education intervention, Acceptance and Commitment Therapy, positive psychology, acceptance, mindfulness

## Abstract

There has been a rapid growth in positive psychology, a research and intervention approach that focuses on promoting optimal functioning and well-being. Positive psychology interventions are now making their way into classrooms all over the world. However, positive psychology has been criticized for being decontextualized and coercive, and for putting an excessive emphasis on positive states, whilst failing to adequately consider negative experiences. Given this, how should policy be used to regulate and evaluate these interventions? We review evidence that suggests these criticisms may be valid, but only for those interventions that focus almost exclusively on changing the content of people’s inner experience (e.g., make it more positive) and personality (improving character strength), and overemphasize the idea that inner experience causes action. We describe a contextualized form of positive psychology that not only deals with the criticisms, but also has clear policy implications for how to best implement and evaluate positive education programs so that they do not do more harm than good.

## Introduction

*We speculate that positive education will form the basis of a ‘new prosperity,’ a politics that values both wealth and well-being* (Seligman et al., 2009, p. 293).

Positive psychology is the scientific study of optimal functioning. It seeks to identify the strengths and skills that enable individuals and communities to thrive. We view positive psychology not so much as a distinct field, but rather a distinct way of viewing the human condition. Positive psychologists do not see people as broken or as having something missing. Rather, they see all people as having the potential to thrive given the right skills, strengths, and social context (Kashdan and Ciarrochi, 2013).

There has been a lot of excitement about bringing positive psychology to schools, a movement that has been called “positive education” or education for “both traditional skills and for happiness” (Seligman et al., 2009, p. 293). However, serious criticisms have been made about positive psychology, criticisms which, we feel, if not addressed, could sink the positive education movement (e.g., Lazarus, 2003a,b; Becker and Maracek, 2008; Ciarrochi and Kashdan, 2013; Foody et al., 2013; Friedli and Stearn, 2015; Purser and Milillo, 2015; Arthur et al., 2016).

These criticisms can be summarized as follows: Positive psychology that is decontextualized is coercive, promotes harmful emotion regulation strategies (experiential avoidance), and promotes maladaptive pursuit of positive internal states. We will review in detail these potential problems, which we believe need to be solved in order to preserve what is best in positive education. Then we will present solutions to each of the problems and make policy recommendations on how to implement and evaluate the proposed solutions.

## Focusing on Content Versus Context

Positive psychology is a complex and heterogeneous field, consisting of interventions for promoting resilience, positive emotions, engagement, meaning, curiosity, social connectedness, and many other things (Ciarrochi and Kashdan, 2013; Parks and Biswas-Diener, 2013). In this paper, we make a distinction between two components of positive psychology:

*Content-focused positive interventions:* “Content,” in this article, will refer to the forms of private experience, including thoughts, feelings, images, attitudes, and beliefs. We define content-focused positive interventions as those that focus on altering the content of people’s thinking, that reinforce the notion that a certain way of thinking is inherently good, and that tend to underemphasize or ignore the role of context. These interventions often seek to increase positive mental content (e.g., optimism), or diminish negative mental content (e.g., fear). For example, theorists have argued that positive emotions cause people to “broaden and build” (Fredrickson, 2001), optimism and grit cause people to succeed (Seligman, 2002; Durckworth, 2016), and a lack of positive thinking and/or the presence of negative thinking causes failure and unhappiness (Byrne, 2006; Peal, 2012). These expressions of positive psychology would be considered ‘content-focused’ to the extent that they – in practice – treat optimism, grit, and positive thinking as a universal good, a kind of ‘fuel’ or ‘nutrient’ for achieving high grades, wealth, health, relationship success, and well-being.

It is important to note that even typically contextual interventions can be done in a content-focused way. For example, mindfulness interventions can be treated as a universal way to reduce stress, and administered to every student in school, regardless of their particular history or context. In this instance, giving students mindfulness is seen as being the same as giving them vegetables, milk, or a magical “Buddha pill”(Farias and Wiholm, 2015).

*Context-focused positive interventions*: We propose context-focused positive psychology (CPP) interventions as a way forward. “Context” refers to situational and historical events that exert an organizing influence on behavior” (Hayes et al., 2011, p. 33). More specifically, it refers to influence that comes from the immediate antecedents and consequences of behavior, the historical context (e.g., evolutionary selection and historical period), and the multiple groups and structures within which a person is nested [e.g., social class, culture, family group, friendship group (Bronfenbrenner and Ceci, 1994)]. Our definition of context includes not just external stimuli but also internal stimuli that can exert influence on behavior (Villatte et al., 2016). For example, a university student recalls a supportive elementary-school teacher when facing a tough math exam and increases her study efforts. The recalled memory would be part of the context linked to increasing study behavior.

Elder (1999) provides an example of the importance of historical context. During the great depression, children often developed a sense of competence and responsibility due to a need to contribute to family well-being, and tended to engage in lower levels of antisocial behavior during this period. Parker et al. (2016a) provide a clear example of the importance of considering cultural context. They conducted a large, multi-country study and found that the influence of factors such as positive self-concept on aspirations was weaker in countries where there was more segregation based on achievement. Thus, positive psychology interventions that seek to increase self-concept may have relatively weak influence in these countries.

Focusing on context, rather than content, counters the repeated criticism of positive psychology that it is overly individualistic and has the potential to place sole burden of responsibility on the individual, largely ignoring their individual circumstance. This is especially true if one defines circumstance as “aspects of the environment of individuals that affect their achievement of objectives and for which society or policy makers do not wish to hold the individuals responsible” (Dardanoni et al., 2006, p. 60). A key challenge for positive psychology is to integrate this idea into theory and policy recommendations. Put simply, positive psychology needs to begin taking seriously the tension between individual agency and social structure in the way that sociology has for decades (Bourdieu, 1979).

In summary, context-focused positive interventions treat the causes of behavior as largely residing within the environment. Internal content such as optimism, positive affect, and grit are considered a part of context in the sense that they are derived from historical experience or heredity, and a part of the cause of behavior, but are never viewed as sufficient causes. Even biological states (e.g., parasympathetic nervous system) are part of the context influencing people’s behavior (Sahdra et al., 2015a; Grossman et al., 2016). We will have much more to say about this later, but the key goal of the CPP intervention is twofold: (1) Create environments where young people can choose actions that are personally important and meaningful, and (2) Teach young people skills that help them respond effectively and flexibly to their environment, so that they can reach their full potential.

## Criticisms of Positive Psychology

All our criticisms will focus on interventions that, we believe, place an excessive focus on positive content and too little emphasis on the role of context in influencing behavior. In principle, we believe any positive psychology can incorporate negative content and context. Indeed, within the field of positive psychology, Rashid’s (2015) approach to positive psychotherapy provides one way to integrate mental ill health symptoms with positive character traits and states, and Biswas-Diener and Lyubchick (2013) provide examples of how cultures and subcultures can influence such standard positive psychology interventions as strength spotting. Other positive psychological approaches that have a strong contextual emphasis include the psychological flexibility work of Kashdan et al. (2014) and Kashdan and Biswas-Diener (2015), the self-compassion work of Neff (2011), the personality and strength work of Mcnulty (2008) and Baker and McNulty (2011), and the mindfulness work of Garland and Fredrickson (2013).

Our issue is not with what is theoretically possible, but what is actually emphasized and expressed in positive psychology. Many positive psychology approaches, in our view, have an excessive emphasis on the individual content and on changing internal character strengths and feelings. For example, consider the internal focus of these top selling positive psychology books: Learned Optimism (Seligman, 2011), Positivity: Top notch research on how the 3 to 1 ratio can change your life (Fredrickson, 2010), Positive Psychology: Harnessing the power of happiness, mindfulness, and inner strength (Segal and Leighton, 2016), and How children succeed: Grit, curiosity and the hidden power of character (Tough, 2013). These books don’t theoretically rule out the role of context in influencing behavior. They just emphasize individual characteristics and de-emphasize context to such an extent as to make context invisible. The following metaphor illustrates the problem:

Suppose a nutritionist had written a book about the need to eat porridge every day, and then concluded with a couple of paragraphs about how porridge is not enough but only works as part of a balanced diet. No one would object to such a presentation because most people know about the need for a balanced diet anyway and only require gentle reminders, from time to time, of that truth. Here is the disanalogy, however. There is a powerful agenda in the field of character and character education which misconceives ‘character’ as comprising *exclusively* resilience, grit and other performance virtues (Arthur et al., 2016, p. 8).

We would add: There is a powerful agenda in the field of positive psychology that misconceives the causes of behavior as comprising exclusively internal characteristics such as positive affect, optimism, and character. There is already a common tendency for people to overestimate causes as residing within the individual and underestimate the power of context, an effect called the fundamental attribution error (Jones and Harris, 1967). There is a danger that positive psychology further reinforces this error.

The fundamental attribution error naturally leads policy makers to conclude that if someone is not succeeding, it is because they lack some sort of character strength, rather than lacking a supportive social context. This makes it easier for policy makers to blame the victim of social injustice. It also makes it easier for the victims to turn on themselves, and engage in harmful strategies in order to correct ‘internal problems.’

We will now consider each criticism of positive psychology in some detail.

### Criticism 1: Content-Focused Interventions Are Decontextualized and Coercive

Friedli and Stearn (2015) argue that explaining behavior in terms of positive affect is often decontextualized and coercive. ‘Coercive,’ in this context, means “to compel by force, intimidation, or authority,” especially without regard for individual desire or volition^1^. For example, a child might be singled out as being disruptive in class during an exercise that identifies character strengths. A decontextualized approach ignores factors that might contribute to these behaviors, such as a traumatic history where the child was verbally abused by his father, and therefore his thoughts of strengths co-occur with his memories of abuse. Thus, for this child, at this time, given this context, a character strength exercise may be coercive, i.e., forced on the child despite it not being in his or her best interest. A decontextualized approach also ignores the potential utility of negative content for the child, for example when having a “bad attitude” toward an unfair situation may motivate the young person to change the situation (Becker and Maracek, 2008).

The positive psychology interventions criticized by Friedli and Stearn (2015) instruct people about how to think and feel. Similar criticisms have been recently made about mindfulness-based interventions (Purser and Milillo, 2015), which are increasingly becoming a part of positive psychology (Garland and Fredrickson, 2013). Purser and Milillo (2015) argue that mindfulness has become disconnected from its deeper sociocultural context and turned into a kind of tool for stress reduction, or controlling psychological content. We would argue that Purser and Milillo’s critique only applies to mindfulness interventions with a content focus, as defined above, rather than all mindfulness interventions. Schools can introduce mindfulness with the seemingly benign idea of helping students manage stress or increase academic success. Mindfulness then becomes a universal panacea to cure all student problems. Once again, this approach frames mindfulness as useful, regardless of context, and frames the problem as a deficiency inside the student. From this vantage point, students are assumed to be stressed, not because they are getting bullied or ignored, but because they lack certain skills.

Coercion is a real possibility in a school context, where adults have substantially more power than young people. There is a risk that students will engage in activities that are not in their best interests. In such instances, they may also experience a lowered sense of autonomy. Research is clear that when young people do not feel autonomous, they demonstrate a diminished motivation to learn and to persist at difficult tasks (Grolnick and Ryan, 1987; Vanteenkiste et al., 2004).

### Criticism 2: Content-Focused Interventions Do Not Deal Adequately with Negative Affect and Experiential Avoidance

Positive psychologists have argued that psychology has put too much emphasis on negative affect, mental illness and weakness, and too little emphasis on positive affect (Seligman and Csikszentmihalyi, 2000). Content-focused interventions seek to increase the frequency of positive emotions, such as feelings related to being grateful, optimistic, loving, confident, and strong. In contrast, context-focused interventions seek to increase the frequency of valued and vitality-giving behavior. Context interventions recognize that negative experience is also part of a meaningful life and that attempts to avoid such experience are likely to be ineffective in the long run.

We will now argue that content-focused interventions risk being misguided, and that it is not in the best interest of society to minimize talk about negative feelings and states. That is, positive psychology cannot ignore issues related to experiential avoidance and negative affect (Lazarus, 2003a,b).

Experiential avoidance involves the attempt to reduce the intensity or frequency of unpleasant inner experiences such as anxiety, self-doubt, and distress (Hayes et al., 2011). A plethora of research suggests that experiential avoidance is often an ineffective emotion regulation strategy and often detrimental to health and well-being (Chawla and Ostafin, 2007). Lab based studies have suggested that direct attempts to suppress thoughts often leads to a paradoxical increase in those thoughts (Wegner et al., 1993). Correlational research suggests that individual differences in experiential avoidance are associated with just about every known psychopathology, including depression, post-traumatic stress disorder, social phobia, panic disorder, substance use, and eating disorders (Hayes et al., 2006; Kashdan et al., 2006). Experimental research suggests that emotional suppression and pain suppression are ineffective strategies, leading to increased activation of the cardiovascular system (Gross and Levenson, 1997) and slower recovery from pain (Cioffi and Holloway, 1993). Finally, interventions that work to counter experiential avoidance–including Acceptance and Commitment Therapy and exposure therapy–have been shown to be effective in treating a wide range of conditions (Feske and Chambless, 1995; Hooper and Larsson, 2015; Tjak et al., 2015). Whilst most research has been conducted in adults, adolescent research suggests that experiential avoidance is linked to alexithymia, emotion regulation problems (Venta et al., 2013), anxiety, somatization, behavior problems, and academic struggles (Greco et al., 2008).

There are a number of reasons why experiential avoidance tends to fail. First, distress and valued behavior are often intertwined. We often experience fear of failure when we take on new challenges. We often experience fear of rejection when we try to make new social connections. Thus, if we seek to avoid negative inner experiences, we often also end up avoiding valued activities. A second problem with experiential avoidance is that it requires sustained energy to monitor for the to-be-avoided experience and sustained effort to actually avoid it when it occurs. The energy put into experiential avoidance is often wasted and might be better used for valued activities. Third, experiential avoidance limits our ability to receive information from our bodies, such as that conveyed by emotions and sensations. Anger lets us know that we believe that an injustice has occurred. Fear lets us know that there is something in the future that might be harmful to us. Sadness lets us know that we have lost something valuable. If we avoid inner experience, then we lose a valuable source of information. Research indicates that adolescents who struggle to identify and use their emotions as information are more likely to have eating disorders (Sim and Zeman, 2004), difficulties in developing social support (Rowsell et al., 2016), and difficulties in maintaining mental health and well-being (Ciarrochi et al., 2008).

There is another reason why positive psychology ought to pay attention to the negative. Even the most positive of positive psychology messages can be verbally transformed into a rule that builds experiential avoidance (Foody et al., 2013). Consider the idea that optimism and confidence are important to success. Knowing this, it is easy for people to infer that, if they did not succeed, it was because they lacked optimism and confidence, that they are in fact pessimistic and insecure. Similarly, young people might be taught that a ‘good attitude’ will help them succeed at school. From such seemingly benign ‘words of wisdom’, they might infer that they failed because they had a bad attitude. These forms of reasoning may suggest to young people that negative internal experiences are harmful and should be avoided at all costs.

### Criticism 3: Content-Focused Interventions Promote Harmful Experiential Attachment

Both content and context focused positive interventionists agree that happiness is important to people and a valid purpose of intervention. However, they disagree about how happiness is achieved. Content focused approaches seek to directly increase positive feelings and evaluations. For example, these approaches may utilize mindfulness, loving-kindness, gratitude diaries, humor, and positive affect inductions to improve the relative ratio of positive to negative emotions. They teach that positive emotion should be valued for their own sake and for their power to increase positive behaviors, such as exploring, creating, and building social networks (Fredrickson, 2001; Fredrickson et al., 2008). In contrast, context focused interventions promote flexible, value-consistent behavior, and treat positive states as a frequent result of that behavior. Typically, context focused approaches make no direct attempt to increase positive states (Ciarrochi and Bailey, 2008).

The contrast between the two approaches can be phrased as a question: If you want to make people feel more positive, should you encourage them to pursue positive emotions directly (content focused intervention) or should you encourage them to engage in personally valued activities, even when those activities don’t always produce positive affect (context focused)? We argue the latter is the key to real growth. We turn now to several lines of research that suggests content-focused approaches can be problematic.

Research is beginning to show that encouraging people to pursue happiness has a dark side (Gruber et al., 2011; Tamir and Ford, 2012). For example, placing a high value on feeling happy is associated with lower emotional well-being, higher depression, and greater loneliness (Mauss et al., 2011, 2012). Other research suggests that pursuing happiness is more unhelpful in some contexts than others. For example, higher preference for ‘useful’ emotions (e.g., happiness in collaboration and anger in confrontation) has been associated with greater social support, academic functioning, and health (Tamir and Ford, 2012).

Much research is correlational and cannot provide evidence for whether pursuing happiness leads to lower well-being or lower well-being leads to increased pursuit of happiness. However, other experimental evidence suggests that it is the pursuit of happiness that is problematic. For example, Schooler et al. (2003) instructed one group of participants to make themselves as happy as possible while they listened to music, and gave no instructions to a control group. Participants who tried to make themselves feel happy reported worse mood than those who were given no instructions. In another experimental study, participants were presented with an article arguing for the benefits of happiness, or an article not mentioning happiness. Then all participants watched a happy film clip. Those who were taught to value happiness reported less happiness after the film than the control participants (Mauss et al., 2012).

Ford and Mauss (2014) offer several interrelated explanations for why attempts to directly increase the frequency of positive emotion may undermine happiness. First, encouraging people to pursue happiness may make them set unrealistic standards for happiness and lead to disappointment. Second, people may not know what makes them happy, and may engage in activities that seem to intuitively lead to happiness, but don’t. For example, encouraging people to pursue happiness may lead them to buy things rather than give things away, although it is the latter that is more tied to happiness (Kasser et al., 2014). Third, encouraging people to pursue happiness leads them to monitor their internal states, and this active monitoring may change their experience. People may keep checking in to see if they are happy, rather than being fully immersed in the present moment.

Recent research on non-attachment suggests other explanations of why pursuing happiness may be ill-advised, and that the problem may extend beyond happiness to the pursuit of all positive states. Non-attachment is defined as a flexible, balanced way of relating to one’s experiences without clinging to them or pushing them away (Sahdra et al., 2010, 2016). From a non-attached perspective, positive and negative states are seen as fleeting phenomena in a dynamic interconnected web of internal experiences, inextricably linked to ever-changing contextual/situational factors. The flip side of a non-attached perspective is experiential attachment, which implies being ‘hooked’ to positive (or negative) states, that is, treating them as solid entities that one possesses. In this theoretical framework, reinforcing the pursuit of positive states may unintentionally make these states seem like ‘possessions’ that one must have and cling to. Unfortunately, these particular ‘possessions’ must be repeatedly lost. The transitory nature of positive feelings and life ensure that. In addition, reinforcing the pursuit of positive states may cause people to become attached to their own positive feelings and devalue the feelings of others (Sahdra et al., 2015b). That is, it may make them more selfish.

Research confirms the downsides of experiential attachment. For example, people high in experiential attachment, that is, those who have a higher tendency to cling to positive states, compared to their non-attached counterparts, have higher depression, anxiety, and suicide rumination (Sahdra et al., 2010; Lamis and Dvorak, 2013). Consistent with the theory that experiential attachment to self-enhancement feelings and thoughts is a barrier to helping others, research has found that experientially attached individuals are less generous toward others (Sahdra et al., 2010), and less open-minded (Sahdra and Shaver, 2013). They also exhibit lower life satisfaction and are less effective in managing time, handling social situations, achieving important goals, changing their point of view in the face of contradictory evidence, facing challenges with a sense of calm and mental balance, and trusting themselves to fully engage with the present task (Sahdra et al., 2016). In the youth literature, research has shown that adolescents who are high in experiential attachment are less likely to engage in prosocial helping behavior as observed by peers (Sahdra et al., 2015a). Taken together, these findings suggest that experiential attachment to positive states can interfere with effective living and prosociality and lead to poor mental health.

### What about the Evidence That Content Interventions Work?

Research shows that positive constructs like optimism, positive affect, and ratio of positive to negative events predict positive outcomes (Garland et al., 2010; Diehl et al., 2011; Ciarrochi et al., 2015). Further, positive education interventions have been shown to produce benefit (Waters, 2011). If there is evidence of benefit, why then bother with these criticisms? Don’t these positive outcomes prove that the criticisms are wrong?

We have three replies to this argument. First, demonstrating that optimism, self-confidence, and positive affect are correlated with beneficial outcomes does not indicate *if and how* interventions should go about increasing these internal experiences. For example, we know that self-esteem predicts positive social development (Marshall et al., 2014). Should we therefore teach everybody that they are special and important? Baumeister et al. (2003) argue that such a self-esteem boosting approach might increase antisocial behavior, as young people come to believe that their worth is not contingent on what they do. Should we teach people to value positive affect, because it “causes” creativity? Such an approach might actually lead young people to focus excessively on generating positive affect and distract them from the creative task.

Second, even if positive education programs have been shown to work, there is still little evidence as to why they work. Many evaluation designs involve administering a complicated positive psychology package to students. Such packages typically include many different components, some of which could be inert or even harmful. In addition, many educational research designs are not gold-standard, randomized control, which involves comparing the intervention group to an active control (Haynes et al., 2012). Weaker designs do not allow us to rule out the possibility that a positive education program is effective simply because of placebo effects or because it increases the amount of attention given to students (Adair, 1984).

Our criticism of the package approach does not just apply to positive education interventions. Indeed, this problem is common throughout much intervention research, and more than a decade has passed since Rosen and Davison (2003) called for researchers to focus on empirically supported processes rather than packages (Rosen and Davison, 2003). There has been an increase in process focused research recently (Hayes et al., 2006; Levin et al., 2012), but there is still much more to be done before we have a strong idea of what components of an intervention are more or less effective. Thus, our first policy recommendation is for more research focused on establishing empirically supported processes.

## Suggested Government Research Agenda

The three criticisms described above do not prove that positive content interventions necessarily lower ones feeling of autonomy (from coercion), or increase one’s tendency to engage in experiential avoidance or attachment. However, the criticisms and the evidence behind them do raise the real possibility of unhelpful processes and outcomes. Given the first rule of intervention research should be “do no harm,” we believe one should proceed cautiously with content focused interventions, by contextualizing them as described in the next section, and by continuing to conduct research examining when intervention processes are more or less helpful.

Research focused on entire intervention packages can never tell us the extent that content-oriented or context-oriented interventions within that package are useful. Governments need to support research that seeks to establish empirically supported processes of change, rather than empirically supported packages (Rosen and Davison, 2003). There are several ways to accomplish this. First, researchers can utilize mediation analysis to examine whether the purported processes targeted by the intervention are actually changing as a result of the intervention and whether these changes in turn produce downstream benefits. For example, research suggests that Acceptance and Commitment Therapy increases psychological flexibility and mindfulness-based cognitive therapy increases self-compassion, and changes in both of these processes predict changes in well-being (Ciarrochi et al., 2010; Kuyken et al., 2010). Second, instead of comparing interventions to neutral control groups, researchers should use dismantling designs, which involves comparing a full positive education intervention to smaller interventions that consist of components of the full intervention (Jacobson et al., 1996). For example, one could compare an intervention that seeks to increase positive affect and prosocial behavior with one that focuses on increasing either prosocial behavior or positive affect. This design allows one to determine the extent that each of the components adds value. In some cases, we might discover that the full program is no more effective than a subcomponent of the program (Jacobson et al., 1996).

Even the notion of ‘effectiveness’ needs to be contextualized. We need to know the cost-effectiveness of a particular intervention, in a particular context, at a particular time (Hollands et al., 2013). Interventions can be shown to produce statistically significant benefit but not be cost effective in some contexts, due to either small effect sizes or costly implementation, or both. For example, let’s say a universal positive education intervention costs approximately $14000 a student per year and produces a short term, 0.1 standard deviation improvement in life satisfaction. Is this money well spent? Or, would it be better spent by providing students with an intervention that reduces dropout rate (Hollands et al., 2013), given one estimate suggests that each young person who is prevented from dropping out will save the government 1 million U.S. dollars in both taxpayer losses and total social burden (Levin and Belfield, 2007)? The answers to these questions are probably, it depends. For example, in a disadvantaged school, where dropout is common, program that creates inclusive contexts to prevent drop out might be better than a program that promotes positive character.

## Suggested Policy Initiatives

Contextual positive psychology (CPP) school interventions can have infinite targets, ideally situated in both the student’s own psychology and their external environment. For example, interventions can increase the extent that school’s provide an autonomy supportive versus coercive environment (Vanteenkiste et al., 2004), can increase skills and well-being of parents and teachers (Duineveld et al., under review), can ensure that at risk students are provided with adult mentors (Sinclair et al., 2005), can reduce the extent that students are streamed into different academic pathways (Parker et al., 2016b), and can develop government policies that emphasize the relative importance of academic achievement over social and emotional learning (SEL; Durlak et al., 2011). Here, we will narrow our discussion to a contextual intervention framework that has clear policy implications and is meant to deal with the main criticisms of positive psychology, namely that positive psychology is coercive, excessively focused on content, and excessively individualistic.

We now describe DNA-V, a contextual model of flourishing (Hayes and Ciarrochi, 2015). This is just one possible vision of a CPP approach grounded in contextual behavioral science–a scientific and pragmatic way of understanding behavior, finding solutions to human problems, and promoting human growth and development. Our approach arises from five streams of knowledge: Positive Psychology (Kashdan and Ciarrochi, 2013), Acceptance and Commitment Therapy (Hayes et al., 2011), Relational Frame Theory (Hayes et al., 2001), evolutionary science and learning (Wilson et al., 2014), and operant theory (Skinner, 1969). We should note that the six policy recommendations we make below do not depend on the DNA-V model, but rather on the best available evidence. Other models may similarly capture these six principles.

**FIGURE 1:**
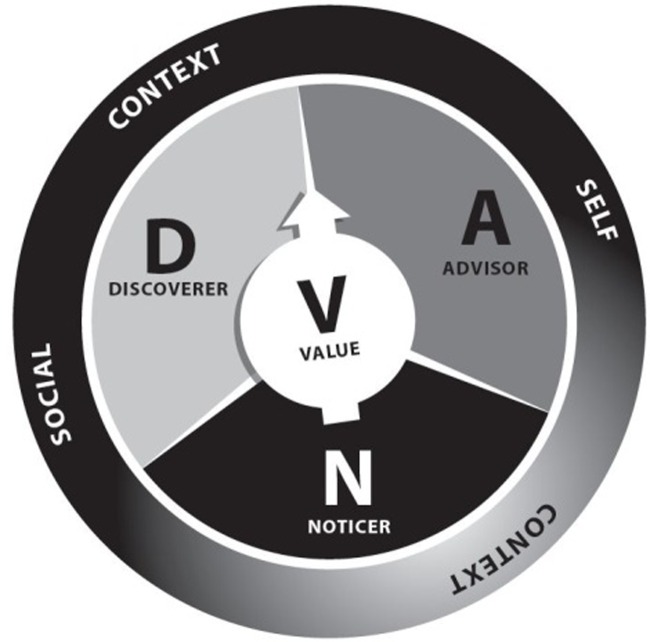
**The DNA-V model of thriving**.

DNA-V is a model that is intended to: (1) suggest a concrete contextual intervention, and (2) provide a framework for understanding the active ingredients in youth interventions, including mindfulness-based interventions, SEL programs, and cognitive behavioral interventions (Hayes and Ciarrochi, 2015). D, N, and A stands for three functional classes of behavior that are embedded inside contexts (self and social) and are optimally used in the service of building values and vitality (“V”; **Figure 1**). Discovery (D) refers to trial and error behavior that functions to broaden and build skills, resources, and social networks. Noticer (N) refers to attending behavior that functions to increase awareness of inner and outer experience. And advisor (A) refers to verbal behavior that functions to save people from needing trail and error learning, thereby navigating efficiently and safely through the world. The model guides the practitioner to develop young people’s skilled use of D, N, and A, to improve their ability to flexibly shift behaviors, depending on what they value and what the situation allows and demands. We will map each of these processes clearly to the wider research literature below.

DNA-V is a positive psychology intervention in that it: (1) seeks to promote thriving, rather than cure disorder, and (2) assumes that people’s apparent dysfunctional behavior is an adaptation to a specific context or niche, and is not due to some character defect or problem. However, DNA-V differs from content-focused positive psychology approaches in that it does not consider any inner experience *a priori* as good or bad; instead it considers how the inner experience connects to overt behavior and functions in a specific environment. The goal of DNA-V, as we will see below, is not to increase optimism or positive affect, but rather to increase behavior that helps a person flexibly adapt to their environment in a way that is value consistent and helps them to reach their full potential. All psychological states – optimism or pessimism, positive or negative affect – may occur in the presence of valued action and are not, by themselves, viewed as causes of that action.

DNA-V does not seek to create something completely new. Rather, it builds on past successful intervention research, as described below, and seeks to implement mindfulness and positive psychology into best practice social and emotional learning (SEL) procedures (Ciarrochi and Hayes, 2016). A recent meta-analysis suggests that teacher-led SEL programs can result in improvement in social and emotional skills, attitudes toward the self and others, positive social behaviors, conduct problems, emotional distress, and academic performance (Durlak et al., 2011). Importantly, programs that followed best practice SEL procedures were associated with better outcomes than those that did not follow best practice (Durlak et al., 2011).

DNA-V and best practice programs follow the SAFE framework (Durlak et al., 2011; CASEL, 2015). Sequenced: The program utilizes a series of connected and coordinated activities to foster skills development. Active: The program utilizes active, experiential forms of learning. Focused: The program is focused on developing social and emotional skills. Explicit: The program targets the core SEL skills identified by CASEL, which are self-awareness, self-management, social awareness and relationship skills, and responsible decision making (CASEL, 2015). Mapping DNA-V to SEL is beyond the scope of this paper, but the interested reader is referred to Ciarrochi and Hayes (2016). We now suggest six concrete recommendations based on DNA-V, one possible framework for CPP.

### Recommendation 1: Create Contexts That Empower Young People to Clarify What They Value and Choose Value-Consistent Actions

The DNA-V model is designed to help people flexibly respond to their environment and live in a way that is consistent with their values and builds vitality. As can be seen in **Figure 1**, values are at the heart of the model, and DNA-V interventions are guided by a context of values. We define values here as qualities of action that are chosen because they are personally important and meaningful (Hayes and Ciarrochi, 2015). Examples include connecting with others, challenging oneself and learning, embracing the moment, giving to others, being physically active, and caring for oneself (Thompson et al., 2008; Hayes and Ciarrochi, 2015).

The DNA-V model is driven by the notion of workability, which is defined in terms of a person’s values and personal context. A behavior is said to “work” for an individual if, in a particular context, it helps that person build value and vitality. What works for one person in one context may not work for another person, or may not work for that same person in a different context. The focus on value and workability undermines coercion, in that it is the young person that ultimately determines what is valued and what works, not the adults surrounding the young person. Values interventions help young people clarify what they value and provide environments that are workable, that is, that allow and encourage young people to engage in behaviors that are personally important and meaningful. These environments have sometimes been termed ‘autonomy supportive’ and the evidence for their benefits is clear (Reeve et al., 2004).

There is also clear evidence for the benefits of value clarification and affirmation for health, education, and relationships (Cohen and Sherman, 2014). For example, multiple studies have shown that getting young African American and Latino students to write about their most important values improves their grades and reduces the racial achievement gap (Cohen et al., 2006; Sherman et al., 2013). Other research suggests that value affirmation can reduce physiological responses to stress (Creswell et al., 2005) and increase willingness to experience pain in the service of value (Paez-Blarrina et al., 2008).

### Recommendation 2: Help Young People to Navigate Their Context with Language

In the DNA-V model, the advisor (A) is a metaphorical name for verbal behavior, or our “inner voice.” It includes beliefs, judgments, evaluations, and making sense of ourselves, others, and life. Advisor is like a GPS system; its function is to guide us efficiently and help us avoid having to engage in trial and error experience for each event. For example, as one of us writes this section of the paper, her advisor is providing a running commentary on her past writing efforts, self-judging if the current writing is the sort of writing that is appropriate and ‘good’ for an academic journal of this standing. The idea of the advisor, in some form, appears in every form of positive psychology and cognitive behavior therapy, in the form of interventions that seek to improve problem solving (Nezu and Nezu, 2001), or to alter the form or function of beliefs, schemas, self-concepts, hope, optimism, self-efficacy, and other verbal evaluations and predictions about the self, others and life (Ellis and Harper, 1975; Young, 1990; Beck, 1995; Snyder, 2002; Seligman, 2004).

Sometimes the advisor provides commentary that is positive and sometimes negative. In a contextualist approach, we don’t assume that advisor behavior is defective if it is generating pessimistic predictions or self-doubt. We ask the question, “What is the consequence of this person listening to their advisor? Does it feed vitality and values? Or does it lead to value inconsistent behavior?”

These questions invoke use of our key tool of ‘functional assessment’ (Ramnero and Torneke, 2008). There are times when pessimism and self-doubt are called for and useful. For example, in one context, self-doubt may be an indication that a person is not fitting into their social world, and it may motivate the individual to build social ties (Leary et al., 1998). In another context, self-doubt might play a causal role in a young person avoiding social contact, and may, consequently lead to a failure to develop social supports (Marshall et al., 2014). This contextual approach overcomes the criticisms of content interventions, because it never tells a person what they ‘should’ think or feel. Instead the main skill is to help people notice when the advisor is helpful, at which time it should be listened to, or unhelpful, at which time one can shift to another behavior to find a more helpful way forward. Helpful here is defined by the person in the context of their lives and values. Helpful and unhelp can never be decided based on content alone, i.e., positive words are not always helpful.

Having identified a functional class of behavior, namely responding to language based rules derived from formal and informal educational experiences, we can then set up educational opportunities to learn advisor behaviors that are flexible and helpful. For example, we might help young people to develop functional hope by setting up opportunities for them to experience success or mastery. We might teach them that success is about acting according to values and not about always getting the ‘right’ outcome. We can also discourage them from relating experiences of failure to the self; as when the thought, “I am a failure” becomes, “This time my efforts did not work out.” However, even as we seek to promote hope, we can teach young people to hold the hope lightly. For example, we can teach them that they don’t ‘need’ to have feelings of hope, at any particular time in order to work toward their valued goals. They learn that sometimes their advisor will say discouraging, hopeless things, and when this happens they can “step out of” their advisor and shift to noticer or discoverer skills (described below). Or, if a situation is actually difficult and low hope is justified, the young person may choose to listen to the advisor and try to adapt to or change the situation.

There is clear evidence for the benefits of ‘listening to a helpful advisor,’ or as more typically expressed, having functional beliefs in specific contexts. For example, the ‘advisor’ probably evolved for the purposes of looking out for danger, so negative thought content is normal and often useful. Similarly, some ‘positive’ advisor thought content is useful too: Young people experience better academic and well-being outcomes if they believe that they can accomplish their goals (authentic hope), that they have social worth (authentic self-esteem), and that problems are more of a challenge rather than an overwhelming threat (effective problem orientation; Ciarrochi et al., 2007, 2009, 2015; Leeson et al., 2008; Marshall et al., 2014).

What do we do if young people experience advisor content that is not building life, but orienting the person to escape life, such as when thoughts of hopelessness are associated with suicide attempts? We could teach them to use cognitive reappraisal so that they do think more positively (Beck, 1995). In our model, we would call this ‘working inside the advisor space.’ However, if this is not done contextually, reappraisal can fail to work, and indeed some research suggests that higher use of reappraisal may be associated with increased negative affect amongst adolescents (Brockman et al., 2016).

Using DNA-V, if changing the content of thinking does not work, we can always shift into noticer space. Or to put this more technically, we can use mindfulness and acceptance skills to decouple the link between verbal content and unhelpful action. A recent review found 44 studies that demonstrated a decoupling of inner experience and outcomes (Levin et al., 2015). For example, mindfulness-related processes can help people to experience physical pain and still persist at a task (Gutierrez et al., 2004), experience approach oriented thoughts to alcohol and not engage in hazardous drinking (Ostafin et al., 2012), experience urges to smoke and unhelpful thoughts, and not still smoke (Elwafi et al., 2013; Adams et al., 2014), experience disordered eating cognitions (e.g., fear of weight gain) and not engage in problem eating behaviors (Ferrieira et al., 2011), and experience negative emotion but not engage in avoidance behavior (Wolgast et al., 2011). This evidence suggests that we can enhance valued action without having to change the content of people’s thoughts, or, metaphorically, to change what the advisor is saying.

### Recommendation 3: Help Young People Notice Inner and Outer Experience to Appreciate Their Present Context and Choose

The noticer (N) refers to a functional class of behaviors that allow us to connect with our inner experience and the physical signals coming from the world around us, and allow those experiences to just be messages, without pushing them away or clinging to them. For example, a young girl may be taught noticer skills to normalize her bodily sensations, become aware of her anger, and learn to allow such feelings to come and go without acting on them; she can use her noticer skills to have anger without needing to hit another student. Or a boy may be taught to recognize the stress in his body when he is studying for a tough exam. He might also notice that when this stress “shows up,” he procrastinates by playing video games. The noticer skill allows him to have the stress, recognize what he usually does, and to either choose what he usually does (procrastinate) or choose something new. Most youth interventions contain a component that targets what we call noticer behaviors. This component can be found in interventions that seek to improve mindfulness (Biegel et al., 2009), awareness and acceptance of sensory input (Kolk, 2006; Pollatos et al., 2008; Farb et al., 2013), skill at describing and labeling emotions (Gottman et al., 1996; Durlak et al., 2011; Kehoe et al., 2014), and skill at responding to feelings in an adaptive and non-impulsive way (Neumann et al., 2010; Hayes et al., 2011).

In the DNA-V model, noticer behavior has five important functions. First, it helps us to tune into our body, detect sensations and identify and label emotions. Second, the noticer helps us to map how our actions affect others. Third, the noticer can be used to tune into the world and what it has to offer. Fourth, when we find ourselves stuck inside thinking (i.e., stuck listening to our advisor), the noticer provides a way to reconnect with the physical realm and loosen the grip of judgements, evaluations, and predictions. Finally, noticer skills involve allowing and being non-reactive to inner experience, which helps young people to regulate their behavior in the presence of difficult emotions. Research suggests that all of these skills are linked to adolescent social and emotional well-being (Ciarrochi et al., 2008, 2011; Rowsell et al., 2016).

As we mentioned in the criticism of content-focused positive psychology, mindfulness interventions can become coercive if their primary purpose is to convince people that the problem resides in their internal states (e.g., “the problem is you’re stressed”) rather than external situations (“the problem is that the teacher is yelling all the time”). This problem of coercion is overcome in DNA-V’s use of the noticer, which puts mindfulness into the service of growth – building the person’s values and vitality. There is no expectation in the DNA-V model that everybody ‘should’ be mindful, or that mindfulness is somehow a simple fix for any problem. In addition, mindfulness training in DNA-V does not involve pressuring people to feel more positive or less stressed. Indeed, a core component of DNA-V noticer training involves learning to allow and not react to whatever private experience is present, no matter whether it is positive or negative. Thus, mindfulness practice may lead to increases in stress, as, for example, when young people acknowledge their fear of a bully or of failing a test.

### Recommendation 4: Help Young People Explore in Order to Develop Skills and Resources, and Expand Their Context

We refer to the final major behavioral class in DNA-V as the discoverer. In contrast to the advisor whose function is to free people from trial and error learning, the function of the discoverer is to engage with the world and learn through trial and error how to interact and manipulate it. Trial and error is inherently risky but functionally adaptive. In infancy, for example, learning to walk requires an infant to stand up, fall down, and stand up again, despite the pain. Play is adaptive for children and teaches growth through risk. In the game of peekaboo, the child must learn to have fear and still seek out the hidden person. In adolescence, risk taking, love of novelty and sensation seeking are key characteristics seen across the species and conceptualized as adaptive behavior that helps juveniles test adult behaviors (Shlegel and Barrhy, 1991; Spear, 2004; Ellis et al., 2012). Thus, in adolescence, high skilled discoverers engage with the world in ways that helps them build skills, learn through trial and error, and grow. Low skilled discoverers are impulsive and do not learn by trial and error in ways that lead to growth. Discoverer interventions deliberately map to the evolutionary science behind risk taking, and consider how risk taking maps to adaptation. In this way, we minimize coercion by helping young people discover what risk taking actions help them to build value and vitality, rather than pressuring them to reduce risk taking in all contexts.

Discoverer as intervention maps most closely to the positive psychology idea of broadening and building (Cohn and Fredrickson, 2006; Waugh and Fredrickson, 2006; Garland et al., 2010). It also has similar functions to CBT interventions that encourage people to engage in new behaviors in order to help them discover valued activities (Jacobson et al., 1996) and interventions that focus on function and teach people to track the consequences of their behavior (Hurl et al., 2016). In the realm of measurement models, high discoverer skill can be roughly mapped to adaptive expressions of openness to experience (Headey and Wearing, 1989; Davey et al., 2003; Heaven and Ciarrochi, 2012) and curiosity (Kashdan et al., 2009), and low discoverer skill can be mapped to maladaptive forms of sensation seeking (Roberti, 2004).

Whilst discoverer behaviors map roughly to Fredrickson’s (2001) broaden and build behaviors, they differ in important ways. Fredrickson’s notion is that positive affect leads to broaden and build behavior, and there is indeed evidence for this (e.g., Garland et al., 2010). However, the contextualistic perspective we outline expands this idea by showing that broaden and build does, and indeed often must, occur in the presence of negative affect. Discovery demands trial and error learning and leaving the safety of pre-existing beliefs behind; fear and exploration often co-occur. Exposure therapy illustrates this point and is perhaps the most empirically validated process of treatment in clinical psychology (Feske and Chambless, 1995). Exposure can be seen as a type of broadening and building. If, for example, a young person is afraid of socializing, he may react to that fear by avoiding all social events. He may never experience the joy of waving to classmates in the mall, or being able to join a gym class. Exposure exercises could help this person to experience the fear and interact with others, so that he can broaden and build his possible response to others. Perhaps as he interacts he will start to notice things he did not notice before, such as others smiling back at him. Discoverer skills can help him engage in new things such as physical exercise and talking with friends; all trial and error learning used to broaden and build, irrespective of current emotional state.

### Recommendation 5: Help Young People Take Perspective on Themselves and Others

As seen in **Figure 1**, individual skills (e.g., DNA) are surrounded by higher order skills that we call ‘Self-view’ and ‘Social view.’ These skills are not distinct from D, N, or A. They often involve discoverer (e.g., developing the self/ discovering other), noticer (e.g., noticing self-evaluations/other evaluations), and advisor (e.g., beliefs about self and other). What makes self-view and social view distinct is that they involve perspective taking, or more technically verbal frames involving I-YOU, HERE-THERE, and NOW-THEN. These frames can relate entirely to the self (I-NOW, see how I felt different THEN) or to others (I-HERE feel angry, and YOU-THERE feel sad) (e.g., see Barnes and Rehfeldt, 2013; Stewart et al., 2013).

Self-view refers to people’s ability to flexibly and compassionately view their self-concepts as events that come and go rather than as ‘things’ that fix them. The positive psychology constructs most closely linked to self-view are growth mindset (Yeager and Dweck, 2012) and self-compassion (Breines and Chen, 2012; Marshall et al., 2015). Young people with low self-view skills act as if a self-evaluation like, “I am bad at math” defines their essence, the same way wood might define the essence of a chair. In contrast, young people with high self-view skills are able to take a perspective like, “I (NOW)” see that I (THEN) had the thought “I am bad at math.” This perspective allows young people to experience self-concepts as fleeting experiences, rather than ‘real limitations.’ They learn to disregard or set aside unhelpful self-concepts in order to develop and grow. In this way, we see the sense of self can act as a context that affects how DNA behaviors are used. For example, someone who rigidly clings to the self-concept “I am bad at math” will use DNA behaviors to support this self-concept. For example, they may use reasoning/advisor skills to figure out how to get out of doing math homework. Or they may use discover skills to find find non-math domains where they can develop positive self-concept, and invest all their time and energy in that domain. In this way, they may never discover their true potential in math.

We have thus far focused on individual level interventions, but it is clear that perhaps the most important context for humans is social. Decades of multidisciplinary research in attachment (Bowlby, 1979), psychology (Johnson et al., 2013), animal research (Harlow, 1959), neurodevelopment (Szalavits and Perry, 2010), neuropsychology (Lieberman, 2013), and anthropology (Hrdy, 2009) show that relationships with family and friends are essential to every aspect of our wellbeing. Despite this breadth of knowledge, too often interventions focus on the individual, thereby underestimating the social context. Nowhere is this more erroneous than in adolescence.

In DNA-V, social view points to the influence that social connectedness, relationships and the interdependence of self-with-others has on our ability to use our D, N, and A skills. You may consider this the ‘we view.’ Young people with poor social view skills take negative social interactions personally, and they are unable to step into the shoes of another person and discover that others can and do have different intentions, views, and ways of seeing the world. Social view closely maps to the construct of perspective taking of others, which builds empathy and compassion (Sahdra et al., 2015a; Ciarrochi et al., 2016). For example, a young person with poor social view skills may see another’s intentions as a personal attack, “You deliberately bumped into me” rather than pausing and hearing the other person’s view. Such young people are using their DNA skills for defense rather than for building social connections. Young people with high social view skills are able to adopt a position that “I HERE NOW” is not always the same as “YOU THERE THEN.” They are able to pause and step into another person’s shoes. They learn to reach out, to build relationships. When they do this they learn that we all have our own D, N, and A behavior.

### Recommendation 6: Apply the First Five Recommendations to Social Groups as Well as Individuals

Young people are nested within many groups, including family, school, sports teams, and peer groups. These groups can exert a powerful influence on young people. A CPP approach seeks to increase the likelihood of groups engaging in nurturing and cooperative behavior. We aim to create contexts for prosocial adolescent peer groups that discourage bullying and discrimination, and encourage acts of support and kindness.

Using the DNA-V mode, we can suggest the key elements of a group level intervention. The intervention needs to clarify the group values (V), help the group broaden and build their resources and skills by trying new behaviors (D), become aware of how they feel and how feelings can, if reacted to, undermine or support group effectiveness (N), develop more effective group rules (A), take perspective on themselves within the group (self-view) and take perspective on others (social view). Groups involve a continual trade-off between behaviors that are for the good of the group and behaviors that are for the good of the individual. Thus it is important for any group to support cooperation within the group, while also managing excessive self-interest and supporting cooperation between groups (Wilson et al., 2013). The details of how to achieve this are beyond the scope of this review, but please see Wilson et al. (2013) for more details.

### An Example: How a Contextual Approach Transforms Character Strengths

Positive psychology commonly targets character strengths, so we will focus on it here, while noting that our arguments apply to other internal processes such as positive affect. A content focused intervention treats character as ‘thing’ like with certain properties. Positive traits like optimism are like fuel for achieving outcomes, like magnets for attracting positive people, or are like some magical force that transforms the world. Or, to use an organic metaphor, strengths are like muscles that need to be built up so that we can do the ‘heavy lifting’ of life. This elemental realist approach (Atkins, 2012) implies that character strengths are inherently good and lead to positive outcomes. After all, who would not want more fuel, magnetism, magical force, or muscle?

There is nothing inherently wrong with taking an elemental realist approach but it can give rise to two difficulties. First, as we described under criticism 1, it can lead us to conclude that the responsibility for dysfunction or disadvantage resides within the individual, rather than reciprocally with their social context. This quote provides an example:

*There is no antipoverty tool we can provide for disadvantaged young people that will be more valuable than the character strengths (such as) conscientiousness, grit, resilience, perseverance, and optimism (Tough, 2013, p. 195*).

Are strengths more valuable than providing pathways to meaningful jobs, or financial support for education? Will grit be enough to overcome an abusive parent, unsupportive teacher, restricted social mobility, or high unemployment rates? By extolling the value of character traits, one can take the focus away from genuine problems within the whole system. We often see this in schools, where a student is described as lacking persistence, when in fact the environment is quite hostile and unsupportive.

The second potential problem with the content-approach to strengths is that it can treat having more strengths like grit and perseverance as inherently positive, the way that having more physical strength is a positive (Diener, 2003). This fails to consider the function of the strength behavior, the core feature of a contextualist approach. There is increasing evidence that so called strengths can produce negative outcomes in certain contexts. For example, the ‘strength’ of forgiveness can be associated with decreasing marital satisfaction in some contexts (Mcnulty, 2008), persistence and grit can be focused on unproductive tasks (McFarlin et al., 1984), self-compassion without conscious attempt to improve oneself can lead to worsening marital relationships (Baker and McNulty, 2011), and believing one can improve oneself, a growth mindset, can lead one to be particularly judgmental of others who fail to change (Kammrath and Peetz, 2011). Without context we can forget that there is no strength that is strong or useful in every context, with every person.

The contextualist approach to strengths does not treat any characteristic as inherently good or bad. It does not even treat character strengths as ‘things’ with properties. Instead, character strengths are viewed as patterns of behavior that occur in a particular context, and serve a particular function that may be more or less useful. Persistence is adaptive if the task is valued and/or helps the young person to grow, but not when the task is clearly hopeless. Courage is adaptive when a young person is stepping up to do something important, but maladaptive when a young person is driving too fast to impress his friends.

## Conclusion and Future Directions

More than a decade ago Lazarus (2003b, p. 173) argued that positive psychology “…makes a false dichotomy out of positive and negative rather than integrating them” and “…. presents an almost Pollyanna version of the Garden of Eden notion of the good life and good people ….” “This quote is typical of an attack against a caricatured or straw-man version of positive psychology. From our perspective, most formally designed positive education interventions are unlikely to fall into such simplistic traps. However, we believe that current positive psychology is guilty of a more subtle and pernicious set of errors. We have sought to articulate these errors here by illustrating how they are likely to occur in one component of positive psychology interventions – the components that seek to increase positive inner experience and explain behavior in terms of that inner experience. There is now enough evidence to raise some concerns about this approach. Further research is needed to examine when and how positive content interventions backfire and when they work. We would argue that the effect of these interventions depends on context and the values, histories, and needs of the people involved.

We believe that CPP is needed in schools. CPP presents six contextual recommendations that work against cultural forces that can be harmful to the young person.

First, CPP encourages educators to search for the causes of behavior in the environment *and* its interaction with the individual, rather than assuming the behavior is entirely due to something internal, like a lack of character strength or positive attitude. This goes against the commonly made fundamental attribution error, which involves explaining the behavior of others in terms of internal causes. CPP does not assume that adolescents engage in self-destructive or antisocial behavior because they have a negative attitude or a dopamine imbalance. They are not broken. Rather, their troubled behavior can be understood as an adaptation to their environment. We do not seek to fix them, but rather to create environments where they can thrive. We recognize that their attitudes, feelings, and strengths are an important *part* of the causal stream, part of the individual’s context, but not the whole context. We must ask: “What is it about their environment that reinforces those attitudes, feelings, and strengths? Can we create a better context for them? Can we help them change their own context?”

Second, CPP suggests that we need to create contexts where individuals and groups can work together to discover what they value, and then act according to those values. This goes against the notion that we should inculcate young people with values, or insist that certain minority groups should act like the mainstream group, or insist that some behaviors like going to university “should” be valued. Instead, we need to offer young people enriched environments, where they can experience a wide range of possible ways of living and discover what works for them. We need to also remember that value occurs at multiple social levels, including individual, family, community, and society, and we need to balance individual self-interest with the interests and values of social groups.

Third, CPP suggests that we need to create contexts where we learn to experience thoughts mindfully, as passing events, rather than as literal truths that must be obeyed. In our model, we might say, “You don’t always have to listen to the advisor. Its advice is not always useful.” This approach goes against the dominant ‘context of literality,’ which suggests words should be taken very seriously and are real things that can hurt you or stop you (Hayes et al., 1999).

Fourth, CPP suggests we need to create contexts where negative feelings are noticed and allowed, rather than avoided, reduced, controlled, or eliminated. This goes against many dominant cultural themes, captured in phrases like “don’t worry,” “you don’t want to think about that,” “the frustration you feel is entirely your own creation,” and “don’t have a bad attitude.”

Fifth, CPP suggests we need to create contexts where young people can practice letting go of positive states like joy and contentment. This goes against many cultural themes that advocate that one should “stay positive,” “take a positive attitude,” and “think positive and positive things will happen.” Instead, CPP suggests that we need to learn to let go of the positive states, when holding on to them interferes with what we value. For example, we can’t always feel beautiful, important, loved, and powerful. Clinging to those feelings is characteristic of narcissism (Rhodewalt et al., 1998).

In conclusion, this article has made intervention recom mendations based on the best evidence available, but it is important for policy makers to remember that what is considered ‘best’ is not something that is fixed, but rather is something that evolves based on new scientific discoveries. In a way, this paper suggests a set of evidence-based hypotheses about what is best practice, rather than a set of conclusions. We hope this review prompts policy makers to support further research and training on how we can create contexts where all youth, regardless of race, gender, or cultural background, can thrive.

## Author Contributions

All authors listed, have made substantial, direct and intellectual contribution to the work, and approved it for publication.

## Conflict of Interest Statement

The authors declare that the research was conducted in the absence of any commercial or financial relationships that could be construed as a potential conflict of interest.

## Footnotes

1 www.Dictionary.com, 2016
